# Identification of Triazolopyrimidinyl Scaffold SARS-CoV-2 Papain-Like Protease (PL^pro^) Inhibitor

**DOI:** 10.3390/pharmaceutics16020169

**Published:** 2024-01-25

**Authors:** Sebastjan Kralj, Marko Jukič, Miha Bahun, Luka Kranjc, Anja Kolarič, Milan Hodošček, Nataša Poklar Ulrih, Urban Bren

**Affiliations:** 1 Faculty of Chemistry and Chemical Engineering, University of Maribor, Smetanova Ulica 17, SI-2000 Maribor, Slovenia; 2 Faculty of Mathematics, Natural Sciences and Information Technologies, University of Primorska, Glagoljaška Ulica 8, SI-6000 Koper, Slovenia; 3 Institute of Enviormental Protection and Sensors, Beloruska Ulica 7, SI-2000 Maribor, Slovenia; 4 Biotechnical Faculty, University of Ljubljana, Jamnikarjeva 101, SI-1000 Ljubljana, Slovenia; 5 National Institute of Biology, Večna Pot 111, SI-1000 Ljubljana, Slovenia; 6 National Institute of Chemistry, Hajdrihova 19, SI-1000 Ljubljana, Slovenia

**Keywords:** drug design, protease inhibitor, SARS-CoV-2, papain-like protease, PL^pro^, antiviral design, in silico drug design, CADD, virtual screening, HTVS, structure-based design

## Abstract

The global impact of severe acute respiratory syndrome coronavirus 2 (SARS-CoV-2) and its companion disease, COVID-19, has reminded us of the importance of basic coronaviral research. In this study, a comprehensive approach using molecular docking, in vitro assays, and molecular dynamics simulations was applied to identify potential inhibitors for SARS-CoV-2 papain-like protease (PL^pro^), a key and underexplored viral enzyme target. A focused protease inhibitor library was initially created and molecular docking was performed using CmDock software (v0.2.0), resulting in the selection of hit compounds for in vitro testing on the isolated enzyme. Among them, compound 372 exhibited promising inhibitory properties against PL^pro^, with an IC50 value of 82 ± 34 μM. The compound also displayed a new triazolopyrimidinyl scaffold not yet represented within protease inhibitors. Molecular dynamics simulations demonstrated the favorable binding properties of compound 372. Structural analysis highlighted its key interactions with PL^pro^, and we stress its potential for further optimization. Moreover, besides compound 372 as a candidate for PL^pro^ inhibitor development, this study elaborates on the PL^pro^ binding site dynamics and provides a valuable contribution for further efforts in pan-coronaviral PL^pro^ inhibitor development.

## 1. Introduction

Starting with reports of a novel pneumonia in Wuhan, China, the severe acute respiratory syndrome coronavirus (SARS-CoV-2) is now widespread and responsible for over 4.5 million deaths, impacting the lives of people all around the world [1,2]. SARS-CoV-2, a member of the *Coronaviridae* family and the subgenus Betacoronavirus, represents a positive-sense, single-stranded (+ssRNA) RNA virus, phylogenetically closest to SARS-CoV, which was responsible for the 2002–2004 outbreak [3,4]. Although several vaccines and therapeutic antibodies targeting SARS-CoV-2 have been developed, the biggest drawback of such therapies is the presence of frequent mutations occurring on the Spike protein. Such mutations can render antibodies and vaccines ineffective, creating a need for drugs that target remaining SARS-CoV-2 proteins [5,6,7].

PL^pro^ is one of two proteases present in SARS-CoV-2, with the other one being the main viral protease (3CL^pro^). PL^pro^ is responsible for cleaving two SARS-CoV-2 polyproteins (pp1a, ppa1ab) that contain non-structural proteins (Nsp), which are involved in subsequent viral replication steps [8]. Besides its primary function as a cysteine protease, it exhibits both deubiquitinating and deISGylating functions, which have an impact on the host’s innate immune system [9,10]. This occurs through the removal of ubiquitin and an interferon-stimulated gene (ISG15) from host cellular proteins [11].

**Figure 1:**
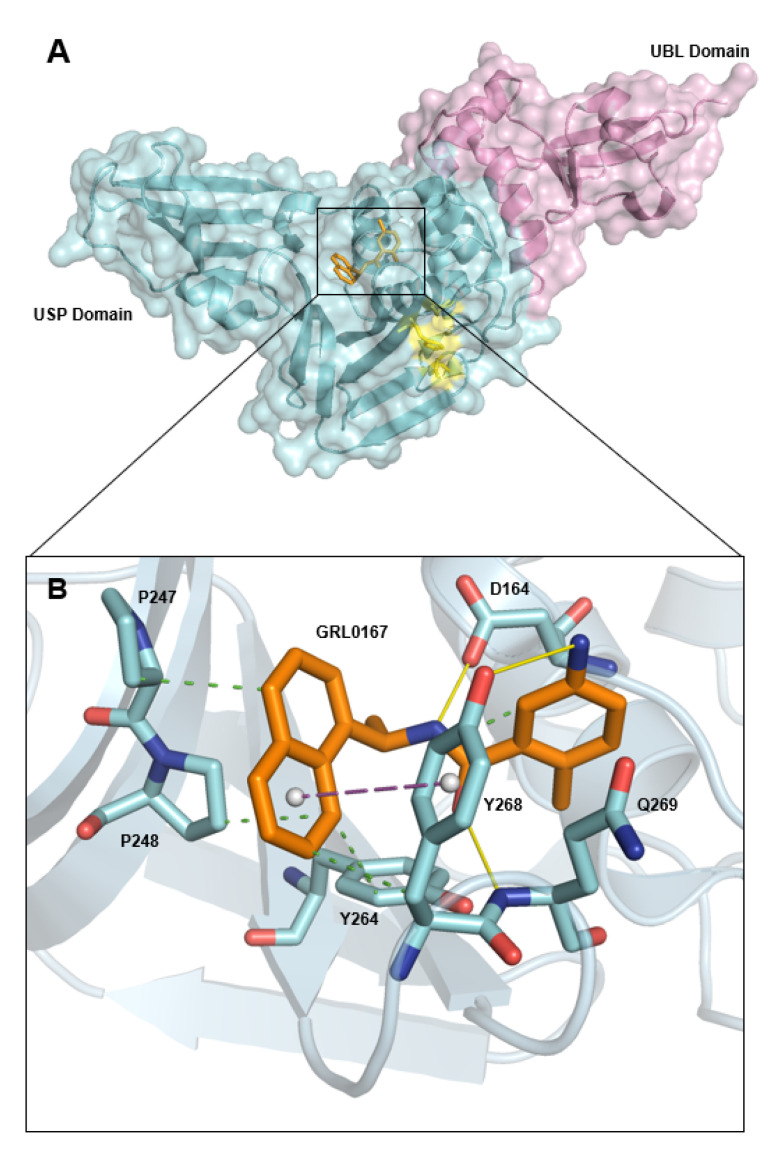
(**A**): Surface and cartoon structure representation of SARS-CoV-2 PL^pro^ (blue, purple, and yellow) in complex with GRL0617 inhibitor (orange). The N-terminal UBL domain (purple) and the C-terminal USP domain (blue) are shown with the catalytic triad labeled (yellow) to depict the proximity of the GRL0617 binding site. (**B**): GRL0617 inhibitor (orange) binding pocket; relevant amino acid residues involved in its binding are labeled and depicted as sticks (blue), with the rest of the protein presented as a cartoon. Important hydrogen bonds are shown by the yellow lines, hydrophobic interactions by dotted green lines, and the π–π interaction by a dotted magenta line [16,18].

SARS-CoV-2 PL^pro^ is composed of two domains: the small N-terminal ubiquitin-like (UBL) domain and the larger catalytic C-terminal ubiquitin specific protease (USP) domain. The larger catalytic USP domain, despite its low sequence identity (~10%) to other ubiquitin-specific proteases, adopts a similar structural arrangement, consisting of three characteristic sub-domains: the thumb, the palm, and the fingers [12,13,14,15]. The thumb is composed of six α-helices and a β-hairpin; the palm of six β-strands; and the fingers of six β-strands, two α-helices, and the zinc binding site (Figure 1) [12]. The catalytic site responsible for the protease activity of SARS-CoV-2 PL^pro^ is located on the interface of the thumb and palm sub-domain, and consists of Cys111, His272, and Asp286 [12,15,16]. This triad cleaves peptide bonds between Nsp1 and Nsp2, Nsp2 and Nsp3, and Nsp3 and Nsp4, yielding three nonstructural proteins: Nsp1, Nsp2, and Nsp3 [17]. The smaller 60-amino-acid-long UBL domain, whose function is unknown, consists of five β-strands, an α-helix, and a 310-helix [12].

Experimental structures of PL^pro^ in complex with either covalent or non-covalent inhibitors are available, providing researchers a great foothold for developing novel PL^pro^ inhibitors [12,16,19,20,21]. The most promising lead so far is GRL0617 (5-Amino-2-methyl-N-[2-(1-naphthyl)ethyl]-benzamide), which has been shown to inhibit not only the proteolytic function, but the deubiquitinating and deISGylating functions of PL^pro^ as well [16]. The crystal structure of the GRL0617 inhibitor bound to the SARS-CoV-2 PL^pro^ protein has been successfully resolved [21] (PDB entry 7CJM). The GRL0617 binding site is formed by a pocket found on the palm region of PL^pro^, and although it is placed in the vicinity, it is not in direct contact with the catalytic triad (Cys111-His272-Asp286). The GRL0617 binding site sits just above the catalytic triad, acting as an anchor point for Nsp proteins and fixating the polyproteins during their cleavage (Figure 1) [20]. Through non-covalent interactions, GRL0617 inhibits SARS-CoV-2 PL^pro^ activity by sterically blocking the binding of the Nsp proteins, ISG15, and ubiquitin to this site [16,22].

**Figure 2:**
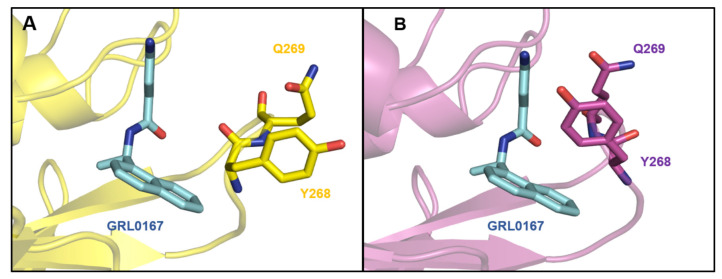
(**A**): The open conformation of the BL2 loop present in the structure with PDB ID 6WX4. (**B**): Closed conformation of the BL2 loop present in the structure with PDB ID 7CJM.

Formation of the SARS-CoV-2 PL^pro^-GRL0617 complex is, in large part, dependent on two hydrogen-bonding interactions: the Tyr268 oxygen with the amino group of the benzene ring of GRL0617 and the backbone amino group of Gln269 with the carbonyl oxygen of GRL0617 [16]. Tyr268 and Gln269 form the BL2 loop, which, upon GRL0617 binding, shifts inwards, deepening the binding pocket. T-shaped π–π interactions, formed by the naphthalene group of GRL0617 with the aromatic residue Tyr268, are formed as a direct result of this inward BL2 loop shifting [22]. The loop exhibits significant flexibility, which results in the formation of the open and closed conformations, with an increased stability of the GRL0617 ligand with the closed conformation (Tyr268 and Gln269 shifted inward) (Figure 2) [16,23]. Other hydrogen bonds important for binding are formed between Asp164 and the amide of GRL0617, as well as between Tyr264 and the carbonyl oxygen of GRL0617, and there are also several hydrophobic interactions that stabilize the complex (details in Supplementary Materials) [16,22].

**Figure 3:**
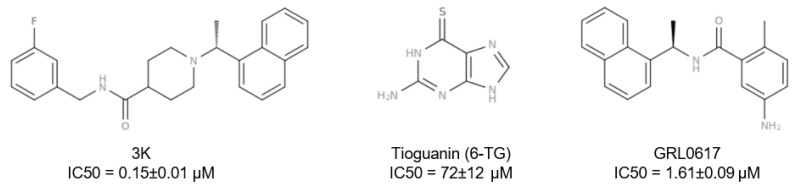
Structural representations of the best-characterized inhibitors of SARS-CoV and SARS-CoV-2 PL^pro^ protein.

The PL^pro^ of SARS-CoV-2 represents an attractive target for drug discovery efforts, with several existing inhibitors already documented. The first non-covalent class of PL^pro^ inhibitors was reported as early as 2008, following the 2003 outbreak of SARS-CoV. These early PL^pro^ inhibitors were naphthalene-based compounds, some exhibiting micro-molar inhibition towards SARS-CoV [24,25,26]. With the SARS-CoV-2 outbreak, the number and diversity of PL^pro^ inhibitors grew further (Figure 3). Despite their greater chemical diversity, from the naphthalene- and piperidine-based 3K to the short peptide VIR251, all inhibitors with solved crystal structures shared the same binding site on the PL^pro^. Inhibition of the deubiquitinating and the deISGylating function of PL^pro^ has, however, not been reported with all inhibitors targeting PL^pro^ [16,19,20,27,28].

As there are currently no viable therapeutic options for targeting the PL^pro^ of SARS-CoV-2, we performed structure-based virtual screening using CmDock. The inhibitory potential of the most promising hit compounds was then tested with in vitro biological assays determining their IC_50_ against PL^pro^. Moreover, the mechanistic insight of the binding mode was explored using molecular dynamics simulations performed in Q6.We successfully discovered a compound exhibiting micro-molar inhibitory properties and showing great potential for further development and optimization. Such small-molecule drugs would act in synergy with vaccination, stopping viral replication, and in turn either treating an established disease or acting as prophylaxis [27]. The performed study draws inspiration from previous work conducted by Jukič et al. on 3CL^pro^ of SARS-CoV-2, as well as Ghosh et al. and Senčanski et al. on SARS-CoV-2 PL^pro^, as these approaches yielded promising results [29,30,31].

## 2. Materials and Methods

### 2.1. Targeted Molecular Library Preparation

**Figure 4:**
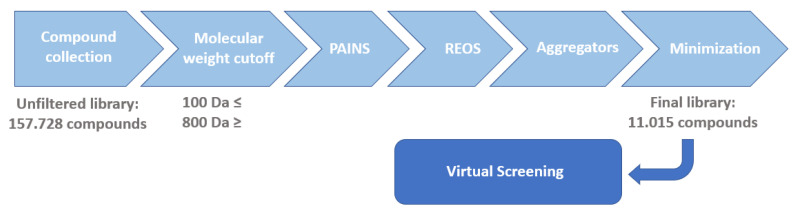
Library preparation workflow for subsequent virtual screening efforts on the SARS-CoV-2 PL^pro^. The final filtered database contained 11.015 compounds before generating other low energy ligand poses for docking.

We collected commercially available libraries marketed as protease inhibitor libraries (APExBIO (Houston, TX, USA), Asinex (Winston-Salem, NC, USA), ChemDiv (San Diego, CA, USA), Enamine (Cincinnati, OH, USA), Life Chemicals (Kyiv, Ukraine), Otava (Ann Arbor, MI, USA), Selleckchem (Houston, TX, USA), TargetMol (Boston, MA, USA)) and expanded the database with all cysteine protease inhibitors and all viral proteases available in the ChEMBL dataset using SQLite. The assembled library was then filtered to exclude small fragments or large molecules, aggregators, and molecules with physio-chemical properties that are problematic in later stages of drug development, i.e., reactive species, optically interfering components, and frequent hitters. This step was completed using KNIME software (4.7; https://www.knime.com; accessed on 20 January 2024) with RDKit nodes integrated in custom workflows [32]. The molecular weight cutoff was set from 100 Da ≤ molecule ≤ 800 Da, with the upper bound chosen based on the fact that peptidomimetic molecules are often above the 500 Da cutoff used with classic rule based filters such as Lipinski’s Ro5 [33]. Filtering of aggregators was performed by comparing the similarity of the Tanimoto coefficients of the library molecules to known aggregators, as well as setting the cutoff of the Tanimoto coefficient similarity at 0.85 and the SlogP value ≤ 3 [34]. Subsequent filtering for REOS and PAINS functional groups with in-house KNIME workflows followed [35,36]. The final step in the targeted library preparation was 10.000 steps of minimization using the MMFF94 force field. Using our in-house KNIME workflows, we were able to reduce the initial library, outsourced from several commercial vendors and ChEMBL, from 157.728 to 11.015 compounds. This was achieved using several property and functional group filters for removal of compounds containing PAINS, REOS, and known aggregators (Figure 4). Geometry optimization, neutralization, and addition of hydrogens were performed using RDKit software for KNIME version 4.2.3 (available at http://knime.org, accessed on 21 November 2021). Molecular docking of the final filtered library ensued.

No conformation sampling was performed at this step, as CmDock uses both stochastic and deterministic search techniques to generate ligand poses [37].

### 2.2. Molecular Docking Using CmDock

The structure of SARS-CoV-2 with GRL0617 bound in the closed conformation was chosen as the starting point for target preparation (PDB ID: 7CJM) [16]. The open conformation of SARS-CoV-2 PL^pro^, with the BL2 loop pointing outward (PDB ID: 6WX4) [20], contains a peptide inhibitor VIR251 that spans from the GRL0617 binding site down to the catalytic triad, where it forms a disulfide bond with C111. To obtain a relevant structure for docking with the open conformation, we cleaved the covalent bond, deleted the peptide inhibitor, regenerated the hydrogen of C111, and minimized the structure using Pymol 2.4.0 (Release 2020-05 Schrodinger, LLC, New York, NY, USA) and CHARMM version 46a2 (details in Supplementary Materials).

The docking programs FRED 4.0.0 (OpenEye Scientific Software, Santa Fe, NM, USA), GOLD (Version 2020.2.0, CCDC, Cambridge, UK) and CmDock (https://gitlab.com/Jukic/cmdock/; accessed on 10 September 2023) were compared using ROC curves to ensure that the optimal docking protocol was used for the full library (details are in Supplementary Materials) [37,38,39]. CmDock was chosen for further docking based on comparable ROC results, working speed, and ease of use. Superimposition of the open conformation (PDB ID: 6WX4) to the closed conformation (PDB: 7CJM) was performed. Both conformations alongside the GRL0617 ligand in the binding pocket were saved as separate structural files. The binding pocket for docking was defined around the GRL0617 ligand [16] and prepared as a sphere of 10 Å around the guide ligand, GRL0617. For docking volume calculation, the CmDock docking package was used (details are in Supplementary Materials). We docked 11.015 compounds of the targeted library to the open and closed PL^pro^ conformation. To select the most diverse scaffolds for developing potential protease inhibitors, an in-house KNIME workflow was used to cluster the top docking results for chemical space occupancy.

### 2.3. Molecular Dynamics Simulation Using Q6

To obtain mechanistic insight into the binding mode, molecular dynamics simulations were run in Q6 (https://www.icm.uu.se/cbbi/aqvist-lab/q/; accessed on 27 December 2023) [40,41]. Ligand–PL^pro^ (PDB ID: 6WX4) complexes from molecular docking calculations were first preprocessed using the Prepare Protein Wizard in the Maestro 12.6 program (Release 2020-4, Schrödinger, LLC, New York, NY, USA). Ligand parameters were determined using Schrödinger ‘s ffld_server and converted to Q6 format using the q_ffld2q.py script from qtools v0.7.0 [41]. The topology files for the MD simulations were created using Qprep6 under spherical boundary conditions. The simulation sphere of 35 Å was defined with Cartesian coordinates at the center of the geometry of all ligands after alignment of all protein–ligand complexes. The same coordinates were used for all simulation systems. Ionizable residues within approximately 32 Å of the spherical center were placed in their ionized states. The side chains of the Asp and Glu residues were treated as negatively charged and the side chains of the Lys and Arg residues as positively charged. In the region between 32 and 35 Å, away from the spherical center, the ionizable residues were mostly treated as neutral moieties. The histidine residues within the sphere were assigned as identified with PROPKA during protein ligand preparation in Prepare Protein Wizard (Release 2020-4, Schrödinger, LLC, New York, NY, USA). In our MD simulations, the following residues were considered to be charged: Arg (82, 140, 166, 183), Lys (92, 105, 157, 274, 297), Asp (76, 108, 164, 286, 302), and Glu (161, 167, 203, 263). Since the number of negative and positive charges in the simulation sphere was equal and all ligands had neutral forms, the net charge was zero, so no neutralization of ions was required. The space not occupied by solutes was filled with randomly oriented TIP3P water. Similarly, the topology of the free ligand was created by constructing a sphere of TIP3P-water molecules around the ligand, again with a radius of 35 Å and the same Cartesian coordinates. The topology files required for the molecular dynamic simulations were created using the Qprep6 subroutine of the Q6 software (v6; https://github.com/qusers/Q6; accessed on 20 January 2024).

MD simulations were performed using the Qdyn6 subroutine of the Q software (v6). The systems were first minimized in a 0.02 ps run at 1 K. Subsequently, the systems were gradually heated from 1 K to 300 K during the three 0.5 ps runs, increasing the time step from 0.2 fs to 1 fs. Production simulations were run at a temperature of 300 K, with a total duration of 40 ns per system and a time step of 1 fs. The OPLS-AA force field was applied for all simulations. Production simulations were performed in two ways. For the first set of compounds, two consecutive 20 ns simulations were performed, while for the second set of compounds, two separate 20 ns simulations were performed, both totaling 40 ns.

### 2.4. Construction of PL^pro^ Expression Plasmid

All amplifications of DNA fragments were carried out via PCR using Phusion high-fidelity DNA polymerase (Thermo Scientific; Waltham, MA, USA). The sequences of all oligonucleotide primers used for DNA amplifications are in the Supplementary Materials. The sequence encoding PL^pro^ was amplified from SARS-CoV-2 cDNA using the primer pair P1/P2. The amplified fragment was assembled into the pMCSG7 expression plasmid by in vivo recombination in *Saccharomyces cerevisiae*. For this, the two fragments of plasmid pMCSG7 were amplified with primer pairs P3/P4 (fragment A) and P5/P6 (fragment B). Additionally, the fragment encoding *S. cerevisiae* 2µ ori and URA3 gene (fragment C) was amplified from the plasmid pYES2.1 using the primer pair P7/P8. The amplified fragments A, B, C, as well as the amplified sequence encoding PL^pro^, were transformed into *S. cerevisiae* INVSc1. The transformants were selected on the synthetic complete agar medium without uracil. The plasmid pURA3_ PL^pro^ assembled in vivo was PCR-amplified with primer pair P9/P10. The fragment C containing the 2 µ ori and URA3 gene was excised from the pURA3_ PL^pro^ using the PstI restriction enzyme. The linearized plasmid was religated with T4 DNA ligase (Thermo Scientific) to generate plasmid p_PL^pro^. This plasmid was propagated in Escherichia coli JM107 in the presence of ampicillin (100 µg/mL) and finally purified using a GeneJET plasmid miniprep kit (Thermo Scientific). The purified plasmid p_PL^pro^ encoded PL^pro^ with His6-tag at the C-terminus downstream of the T7 promoter. The nucleotide sequence of PL^pro^ in this plasmid was determined by sequencing (Macrogen; Seoul, Republic of Korea).

### 2.5. Overexpression and Purification of Recombinant PL^pro^

To produce PL^pro^, the competent E. coli Rosetta 2(DE3) was transformed with p_PL^pro^ and grown in shake flasks at 37 °C in Luria–Bertani broth (Carl Roth; Karlsruhe, Germany) supplemented with ampicillin (100 µg/mL) and chloramphenicol (25 µg/mL). The agitation was set at 250 rpm. At OD600 of ~0.8, ZnSO_4_ (1 mM) was added, and overexpression of PL^pro^ was induced using 0.8 mM isopropyl β-D-1-thiogalactopyranoside (IPTG). After the addition of the IPTG, the cells were incubated at 16 °C for 16 h, with agitation at 250 rpm. The cells were harvested via centrifugation at 4500× *g* for 15 min at 4 °C, and the cell pellets were stored at −80 °C until the PL^pro^ isolation. 

For PL^pro^ isolation, the cell pellets were resuspended in 50 mM Tris (pH 7.4) and 10 mM β-mercaptoethanol (BME), then lysed by sonication. The lysate was clarified via centrifugation at 15,000× *g* for 15 min at 4 °C. The supernatant was collected and combined with (NH_4_)_2_SO_4_ for the final concentration of 1.6 M. After 30 min incubation on ice, the precipitated proteins were pelleted by centrifugation at 15,000× *g* for 15 min at 4 °C and resuspended in buffer A (20 mM Tris (pH 7.4), 500 mM (NH_4_)_2_SO_4_, 10 mM BME). The solution was centrifuged again at 15,000× *g* for 15 min at 4 °C, and the supernatant was loaded onto a HiTrap Phenyl HS column, which was preequilibrated with buffer A. The target protein was eluted by linear gradient from 500 mM to 0 mM (NH_4_)_2_SO_4_ in buffer A. The fractions containing PL^pro^ were combined and passed through a HiTrap DEAE column preequilibrated with buffer A without (NH_4_)_2_SO_4_. The flowthrough was collected and dialyzed in 20 mM HEPES (pH 7.2) and 10 mM BME overnight at 4 °C. The dialyzed solution was then loaded onto a HiTrap SP column preequilibrated with 20 mM HEPES (pH 7.2), 10 mM BME, and 1 M NaCl. PL^pro^ was eluted by linear gradient from 0 M to 1 M NaCl in 20 mM HEPES (pH 7.2) and 10 mM BME. Fractions containing PL^pro^ were combined and stored at −80 °C until analysis. The concentration of isolated PL^pro^ was calculated using absorbance at 280 nm and an extinction coefficient of 45,270 M^−1^·cm^−1^.

### 2.6. In Vitro PL^pro^ Inhibition Assays

The activity of PL^pro^ in the presence of different compounds was determined using the fluorogenic peptide substrate Z-Arg-Leu-Arg-Gly-Gly-AMC (Sigma, St. Louis, MO, USA). The compounds were synthesized using MolPort and dissolved in dimethyl sulfoxide (DMSO) to a final concentration of 4 mM. These compounds were further diluted in 50 mM HEPES (pH 7.2) and 2 mM dithiotreitol (DTT) for the analyses, and the same buffer was used to prepare dilutions of PL^pro^ and substrate. For the initial in vitro screening of inhibitory potencies of synthesized compounds against PL^pro^, the 10 µL 200 µM compounds were mixed with 20 µL 0.4 µM PL^pro^. These mixtures were incubated at 25 °C for 30 min before the addition of 10 µL substrate Z-Arg-Leu-Arg-Gly-Gly-AMC (Sigma). The final concentrations in the reaction mixtures were 50 µM compound, 0.2 µM PL^pro^, and 30 µM substrate. All of the reaction mixtures contained 1.25% (*v*/*v*) DMSO. For the blanks, 20 µL buffer was added to the reaction mixtures instead of the PL^pro^. The reactions were carried out in black 384-well plates. Fluorescence signals were recorded continuously every 15 s for 15 min after addition of the substrate (Spark microplate reader; Tecan; Zürich, Switzerland). The excitation and emission wavelengths were set at 340 nm and 460 nm, respectively.

To determine the concentrations of the selected compounds required to inhibit 50% of the PL^pro^ activity (IC50), 10 µL compounds at different concentrations were mixed with 20 µL PL^pro^ and incubated at 25 °C for 30 min, followed by the addition of 10 µL substrate. The final concentrations in the reaction mixtures were 0.2 µM PL^pro^ and 30 µM substrate. For the blanks, the compounds were mixed with 20 µL buffer instead of PL^pro^ before the addition of the substrate. All of the reaction mixtures contained 25% (*v*/*v*) DMSO. These reaction mixtures were incubated in black 384-well plates at 25 °C. Two hours after the addition of the substrate, the endpoint fluorescence signals were recorded using the microplate reader, with excitation and emission wavelengths set at 340 nm and 460 nm, respectively. All assays were performed in triplicate.

## 3. Results and Discussion

### 3.1. Molecular Docking and Selection of Top Scoring Compounds

With the biological context of our target protein in mind, a set of compounds was chosen for a focused protease inhibitor library which covered as much relevant chemical space as possible with the lowest amount of compounds, thus yielding the best return on computational investment.

Using molecular docking to dock our targeted library, we aimed to discover compounds and scaffolds that could be developed into SARS-CoV-2 PL^pro^ inhibitors. As the designed protease inhibitor library had passed several compound filters, the compounds entering docking were already in accordance with the drug-like paradigm [42]. We selected the binding site of GRL0617 as the target due to the inhibitory, deubiquitinating, and deISGylating functions of GRL0617 when binding to this site. Docking was performed using CmDock (https://gitlab.com/Jukic/cmdock/; accessed on 10 September 2023) on the open and closed conformation of SARS-CoV-2 PL^pro^ (Figure 2). The comparison of docking results from the top 100 scoring compounds across both PL^pro^ conformations showed that the results were diversified between the two groups. Of the top 100 scoring compounds according to CmDock S_inter_ score, 55 were obtained from docking to the closed PL^pro^ conformation and 45 from docking to the open PL^pro^ conformation. The almost equal share among the top scorers shows the importance of using ensemble docking, as proteins in nature may adopt more than one kinetically relevant state [43,44]. The 30 best-scoring compounds were selected for subsequent free energy calculations and in vitro inhibition assays.

### 3.2. In Vitro Assay of PL^pro^ Inhibition

**Figure 5:**
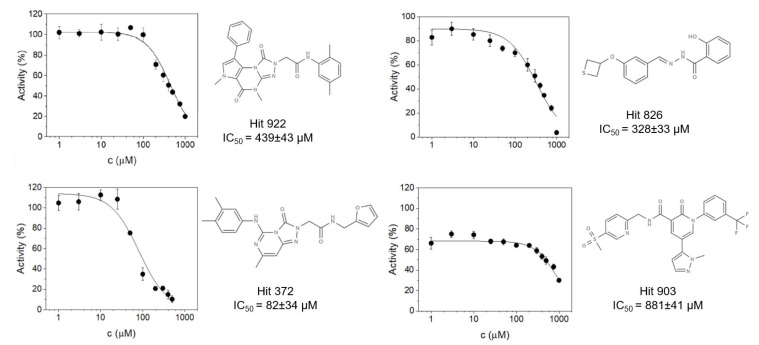
IC50 curves of compounds that exhibited inhibitory properties in in vitro assays.

Half-maximal inhibitory concentration (IC50) in vitro assays were performed to determine the potency of the selected compounds for PL^pro^ inhibition. The assay used the fluorogenic peptide substrate Z-Arg-Leu-Arg-Gly-Gly-AMC to determine the IC50 (Figure 5). Out of the 30 previously selected compounds, 5 exhibited inhibitory potency, with 4 exhibiting IC50 values in the higher micro-molar range. Compound hit 644 was removed from further testing as it exhibited the lowest inhibitory potency, with an IC50 value of 12.1 mM. Three other compounds, Hit 922, Hit 826, and Hit 903, scored in the triple-digit micro-molar range, with IC50 values of 439 ± 43 μM, 328 ± 33 μM, and 881 ± 41 μM, respectively. Of special interest is the compound Hit 372, as it exhibited the best performance, with an IC50 value of 82 ± 34 μM. This result shows great promise, as it is on par with the inhibitor Tioguanin (6-TG), which exhibits an IC50 value of 72 ± 12 μM, and a log unit lower compared to the best inhibitors, such as GRL0617 (IC50 = 1.61 ± 0.09 μM). With regard to further drug optimization, the compound Hit 372 is also interesting, not only due to its low IC50 value, but due to its 1,4,6-Triaza-7-indenone scaffold as well. As retaining scaffolds inside novel compounds as the core structure for further drug design is an efficient way of generating novel, potentially improved compounds, we decided to focus our further computational studies on Hit 372 and its binding energetics and mechanism.

### 3.3. Assessment of Lead-Like Properties for Hit 372

Hit 372 was identified among the docked compounds as having the best activity in in vitro tests, and it shows promise for further development. Its structure is formed by a central pyrimidine-like bicyclic ring system linked on one side by an amide bond to a furan ring, and on the other side to an o-xylene (Figure 5).

**Figure 6:**
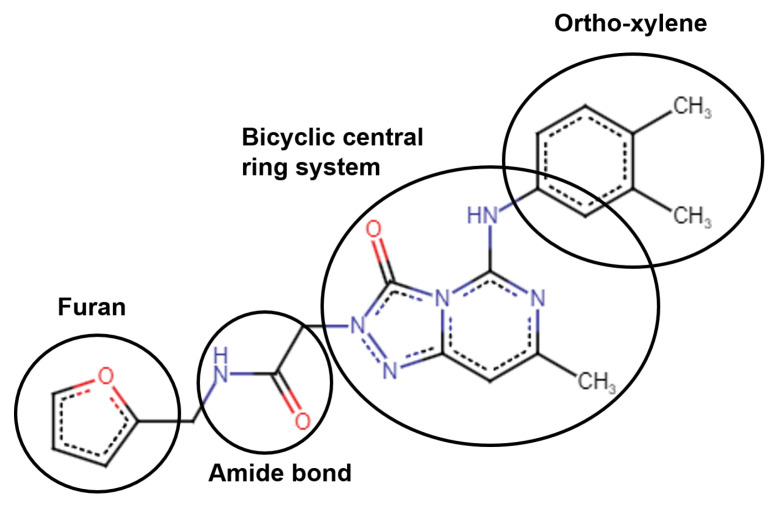
Substructural units of Hit 372 important for PL^pro^ binding.

**Figure 7:**
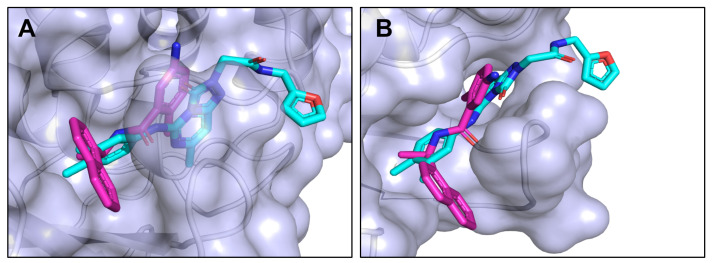
(**A**) Side view of the binding site and inhibitor poses for GRL0617 (purple) and Hit 372 (cyan); (**B**) top-side view of the binding site and inhibitor poses for GRL0617 (purple) and Hit 372 (cyan).

**Figure 8:**
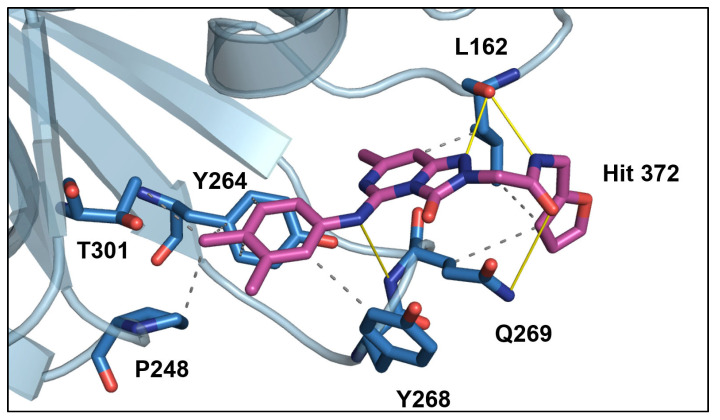
Hit 372 (purple) interactions in the PL^pro^ binding pocket. Amino acid residues relevant in binding are labeled and depicted as sticks (blue), with the rest of the protein presented as a cartoon (light blue). Important interactions like hydrogen bonds are represented by yellow lines, and hydrophobic interactions by dotted lines.

The docked pose of Hit 372 shows that it forms several interactions with the binding pocket of GRL0617. Similarly to GRL0617, the molecule consists of a central ring system that is wedged between the BL2 loop and the rest of the protein. In the case of GRL0617, the central ring is represented by the single ring 4-methylaniline, while Hit 372 is represented by a pyrimidine-like bicyclic ring. Both of the compounds possess a methyl group on the bottom side of the central ring system that seems to anchor the structure. Both compounds possess additional ring systems that play a large role in binding. For GRL0617, it is the signature naphthalene ring, while for Hit 372, it is represented by o-xylene. Both form a vast number of hydrophobic interactions: four for Hit 372 and five for GRL0617. Despite this, the orientation of both rings is different; the naphthalene ring of GRL0617 is wedged between the BL2 loop and the bulk protein compared to the o-xylene, which is perpendicular to it and rests deeper in the pocket (Figure 6). Both compounds possess amide bonds that form hydrogen bonds with the protein. Hydrogen bonds are formed between Hit 372 amide bond oxygen and Glu269 nitrogen, as well as between the Hit 372 amide bond nitrogen and the Leu162 oxygen. However, the relative position of the amide bond is different, as GRL0617 has the bond between the central ring system and the naphthalene ring, whereas Hit 372 has the amide bond on the other side of the central ring system linking with the furan ring (Figure 7). This orientation structurally resembles another PL^pro^ inhibitor, 3K. The Furan substructure is not present with GRL0617, but its analog in the form of a fluorobenzene is present in inhibitor 3K. The furan ring of Hit 372 wraps around the BL2 loop, where it forms hydrophobic interactions with the protein (Leu162 and Gln269) (Figure 8). This, however, is not a stable bond, as the ring is often seen moving during MD simulations, showing potential for improvement through the addition of functional groups.

The key advantage of Hit 372 optimization in future inhibitors is the lack of hydrogen bond interactions with Tyr268, which represents a key bond for the binding of GRL0617 and 3 K. An alcohol group placed on the o-xylene ring could easily interact with the oxygen of either Tyr268 or Asp164. Hit 372 and GRL0617 have four shared interactions with the PL^pro^ amino acids: Pro248, Tyr264, Tyr 268, and Q269. The main candidates, as stated before for optimization, are Tyr268 and Asp164, which both form hydrogen bonds with GRL0617. Another important step would be to optimize the structure of o-xylene for π–π interactions, as they are present with GRL0617, but not with Hit 372. Besides this, Hit 372 is a simple molecule with no problematic substructures, and it is available for purchase through online vendors, making it easily accessible for further research.

## 4. Conclusions

In this study, we identified a novel small-molecule inhibitor of the SARS-CoV-2 PL^pro^ protease—compound 372. The molecule exhibited an IC50 value of 82 μM in in vitro assays using a fluorogenic peptide substrate. Applying molecular dynamics simulations, we confirmed these findings and provided additional insight into the binding mechanism to PL^pro^, showing that the addition of the furan ring and the lack of interactions with Tyr268 and Asp164 facilitate further compound optimization. As such, future research will focus on in-depth analysis of molecular dynamics simulations and understanding of changes in protein–ligand interactions during simulations. Molecular-mechanics-generalized Born surface area (MM-GBSA) calculations will be employed to identify key residues that contribute to both total and residue-decomposed binding free energy. And in addition, the accuracy of the binding free energy calculations will be improved by applying TI (thermodynamic integration) techniques to accurately predict novel inhibitors. As inhibitors for the SARS-CoV-2 PL^pro^ protease are under-researched at the time of writing and compounds such as GRL0617 still the starting points of drug design, our confirmed hits can provide valuable information for future (pan-) coronaviral protease inhibitor design.

## Data Availability

All data are contained within the article and Supplementary Materials.

## Supplementary Materials

The following supporting information can be downloaded at: https://www.mdpi.com/article/10.3390/pharmaceutics16020169/s1. Table S1: Key interactions responsible for GRL0617 binding with SARS-CoV-2 PLpro. Table S2: Table of known inhibitors for SARS-CoV-PLpro. Table S3: MD results for selected docking hits. Table S4: RMSD for selected inhibitors during MD calculations. Table S5: Oligonucleotide sequences used in this study. Figure S1: ROCS curves for conformations of the PLpro protein target.
