# Supplementary material for: Identification of Triazolopyrimidinyl Scaffold SARS-CoV-2 Papain-Like Protease (PLpro) Inhibitor
Source: Pharmaceutics. 2024 Jan 25;16(2):169. doi: 10.3390/pharmaceutics16020169 (PMC10893172; doi:10.3390/pharmaceutics16020169)
Supplement: Supplementary file 1 [file pharmaceutics-16-00169-s001.zip › pharmaceutics-2793231-supplementary.pdf]

pharmaceutics

Article

Supplementary Materials

for

Identification of Triazolopyrimidinyl Scaffold SARS-CoV-2 Papain-Like Protease (PL^{pro}) Inhibitor

Sebastjan Kralj [1], Marko Jukič [1,2,3,*], Miha Bahun [4], Luka Kranjc [4,5], Anja Kolarič [1], Milan Hodošček [6], Nataša Poklar Ulrih [4] and Urban Bren [1,2,3,*]

1 Faculty of Chemistry and Chemical Engineering, University of Maribor, Smetanova Ulica 17, SI-2000 Maribor, Slovenia; sebastjan.kralj1@um.si (S.K.); anja.kolaric2@um.si (A.K.)
2 Faculty of Mathematics, Natural Sciences and Information Technologies, University of Primorska, Glagoljaška Ulica 8, SI-6000 Koper, Slovenia
3 Institute of Enviormental Protection and Sensors, Beloruska Ulica 7, SI-2000 Maribor, Slovenia
4 Biotechnical Faculty, University of Ljubljana, Jamnikarjeva 101, SI-1000 Ljubljana, Slovenia; miha.bahun@bf.uni-lj.si (M.B.); luka.kranjc@nib.si (L.K.); natasa.poklar@bf.uni-lj.si (N.P.U.)
5 National Institute of Biology, Večna Pot 111, SI-1000 Ljubljana, Slovenia
6 National Institute of Chemistry, Hajdrihova 19, SI-1000 Ljubljana, Slovenia; milan@cmm.ki.si
* Correspondence: marko.jukic@um.si (M.J.); urban.bren@um.si (U.B.)

Table of Interactions

Table S1. Key interactions responsible for GRL0617 binding with SARS-CoV-2 PLpro.

Bond type	*Amino-Acid residue (PLpro)*	*Atom number (GRL0617)*	*Distance (Å)*	*Angle (°)*
Hydrophobic interaction	P247	C12	3.73	/
	P248	C14	3.76	/
	Y264	C14	3.67	/
	Y268	C13	3.50	/
	Y268	C20	3.86	/
Hydrogen bond	D164(O)	N2 (amid)	2.82	118.6
	Y268(OH)	N01	3.43	129.54
	Q269(N, backbone)	O7	3.14	146.54
T-shaped π-π interaction	Y268	Naphthalene group	4.73	72.75

Minimization of the Open PL^{pro} Conformation

This was achieved using the CHARMM-GUI Solution Builder for the molecular dynamics program CHARMM[34,57]. Minimization included 50 steps of Steepest Descent (SD) and 500 steps of Adopted basis Newton-Raphson (ABNR), with a further 1 ns equilibration equilibration with the NVT ensemble applied, the temperature was set at 310.15 K using the HOOVER thermostat and the integration time-step set at 1fs. VDW interactions were cutoff between 10-12 Å using the force switch method (VFSWitch). For the calculation of electrostatic interactions, the particle-mesh Ewald summation was used, bonds to hydrogens were constrained using the SHAKE algorithm and the CHARMM 36m force-field was used.

ROC Study

To ensure the best docking protocol was used on the full library, the docking program CmDock (https://gitlab.com/Jukic/cmdock/) was tested on a subset of compounds. This included a random selection of a 1000 decoy drug-like molecules with the average molecular weight of 400 Da, and fifteen known non-covalent inhibitors with structural information deposited on the PDB (Supplementary-Table S2). The structure of the open conformation (PDB ID: 7CJM) and the closed conformation (PDB ID: 6WX4) were selected with all docking calculations, with the binding site prepared separately in each of the chosen programs. Using the CmCavity (https://gitlab.com/Jukic/cmdock/) program, the binding site was defined automatically around the coordinates of the co-crystallized ligand (GRL0617). The generated .grd file was inspected in PyMol to ensure that the binding site has been defined correctly.

Closed PLpro conformation docking cavity:
Total volume 5627.75 A^3
Cavity #1 Size=45022 points; Vol=5627.75 A^3; Min=(-7.5,11.5,-13); Max=(19.5,39.5,10); Center=(4.66316,25.1299,-2.88701); Extent=(27,28,23)
Cav = 45022; total = 45022
Open PLpro conformation docking cavity
Total volume 5557 A^3
Cavity #1 Size=44456 points; Vol=5557 A^3; Min=(-7.5,11.5,-13); Max=(19.5,39.5,10.5); Center=(4.49022,25.1067,-2.85059); Extent=(27,28,23.5)
Cav = 44456; total = 44456
The docking protocol used was defined in the docking parameter file, and was as follows:

```
RBT_PARAMETER_FILE_V1.00
TITLE GRL0617 docking to Plpro

#cavitiy
SECTION MAPPER
    SITE_MAPPER
RbtLigandSiteMapper
    REF_MOL GRL0617_7cjm.sdf
    RADIUS 10.0
    SMALL_SPHERE 1.0
    MIN_VOLUME 100
    MAX_CAVITIES 1
VOL_INCR 0.0
GRIDSTEP 0.5
 END_SECTION

 #restraint
SECTION CAVITY
     SCORING_FUNCTION
RbtCavityGridSF
     WEIGHT 1.0
END_SECTION
#end restraint
```

ROC Curves for docking protocol

Custom KNIME software workflows for ROC curve generation were used for both the open and closed conformation and on all three docking programs, resulting in a total of 6 ROC curves. As stated earlier the results among the docking programs were comparable, and CmDock was chosen based on its speed and ease of use.

Table S2: Table of known inhibitors for SARS-CoV-PLpro

Molecule name IUPAC	Chemical formula/ name	PDB ID	Reference
5-amino-2-methyl-N-[(1R)-1-naphthalen-1-ylethyl]benzamide	C20 H20 N2 O GRL0617	7CJM	Fu et. al
1-(2-methoxyethyl)-2-methyl-3-(pyrazin-2-ylmethyl)benzo[f]benzimidazol-3-ium-4,9-dione	C20 H19 N4 O3 YM155	7D7L	Zhao et al.[58]
N-[(3-acetamidophenyl)methyl]-1-[(1R)-1-naphthalen-1-ylethyl]piperidine-4-carboxamide	C27 H31 N3 O2 Compound S43	7E35	Shan et al.[59]
5-[(carbamoylcarbamoyl)amino]-2-methyl-N-[(1R)-1-(naphthalen-1-yl)ethyl]benzamide	C22 H22 N4 O3 Snyder 495	7JIT	Osipiuk et al.[12]
5-(acryloylamino)-2-methyl-N-[(1R)-1-(naphthalen-1-yl)ethyl]benzamide	C23 H22 N2 O2 Snyder530	7JIW	Osipiuk et al.[12]
3-amino-2-methyl-N-[(1R)-1-(naphthalen-1-yl)ethyl]benzamide	C20 H20 N2 O Snyder441	7JN2	Osipiuk et al.[12]
5-[(E)-(hydroxyimino)methyl]-2-methyl-N-[(1R)-1-(naphthalen-1-yl)ethyl]benzamide	C21 H20 N2 O2, Snyder496	7KOL	Osipiuk et al.[12]
5-[(azetidin-3-yl)amino]-N-[(1R)-1-{3-[5-({[(1S,3R)-3-hydroxycyclopentyl]amino}methyl)thiophen-2-yl]phenyl}ethyl]-2-methylbenzamide	C29 H36 N4 O2 S XR8-89	7LBR	Ratia et al.
5-[(azetidin-3-yl)amino]-2-methyl-N-[(1R)-1-(3-{5-[(pyrrolidin-1-yl)methyl]thiophen-2-yl}phenyl)ethyl]benzamide	C28 H34 N4 O S XR8-24	7LBS	Ratia et al.
5-[(azetidin-3-yl)amino]-N-[(1R)-1-{3-[5-({[(1R,3S)-3-hydroxycyclopentyl]amino}methyl)thiophen-2-yl]phenyl}ethyl]-2-methylbenzamide	C29 H36 N4 O2 S XR8-83	7LLF	Ratia et al.
N-[(1R)-1-(3-{5-[(acetylamino)methyl]thiophen-2-yl}phenyl)ethyl]-5-[(azetidin-3-yl)amino]-2-methylbenzamide	C26 H30 N4 O2 S XR8-69	7LLZ	Ratia et al.
5-(azetidin-3-ylamino)-2-methyl-~{N}-[(1~{R})-1-[3-[5-[[[(3~{R})-oxolan-3-yl]amino]methyl]thiophen-2-yl]phenyl]ethyl]benzamide	C28 H34 N4 O2 S XR8-65	7LOS	Ratia et al.
(1R)-N-[(1H-indol-3-yl)methyl]-N-methyl-1-(naphthalen-1-yl)ethan-1-amine	C22 H22 N2 Jun9-84-3	7RZC	Osipiuk et al.[12]
4-({methyl[(1R)-1-(naphthalen-1-yl)ethyl]amino}methyl)phenol	C20 H21 N O Jun9-72-2	7SDR	Osipiuk et al.[12]
5-amino-N-(naphthalen-1-yl)pyridine-3-carboxamide	C16 H13 N3 O Snyder608	7SGU	Osipiuk et al.[12]
N-(naphthalen-1-yl)pyridine-3-carboxamide	C16 H12 N2 O Snyder630	7SGW	Osipiuk et al.[12]

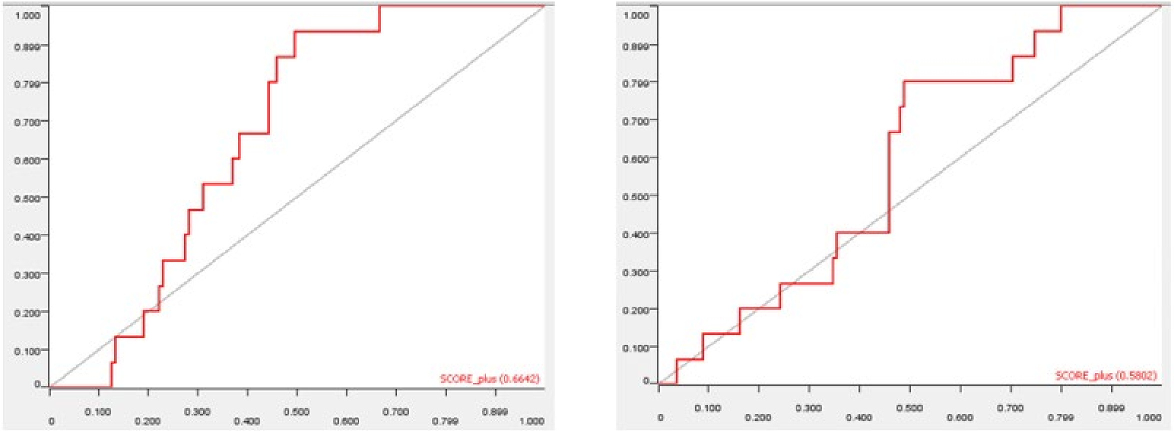

Figure S1: ROCS curves for conformations of the PLpro protein target

Table S3: MD results for selected docking hits

Compound name	Structure
**Hit 903**	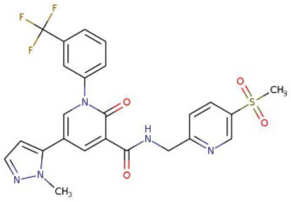
**Hit 922**	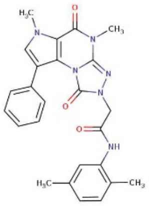
**GRL 0617**	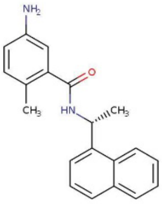
**Hit 826**	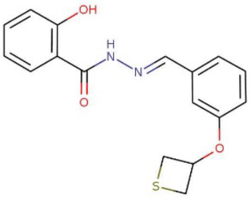
**Hit 372**	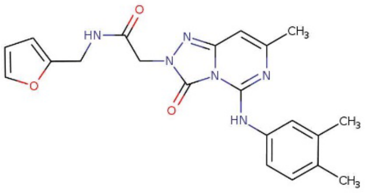

Table S4: RMSD for selected inhibitors during MD calculations

Compounds ID	Average RMSD 1 ± SD[Å]	Average RMSD 2 ± SD[Å]	RMSD protein	RMSD ligand
**Hit 903**	1.313 ± 0.208	1.316 ± 0.105	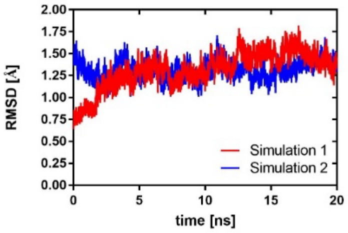	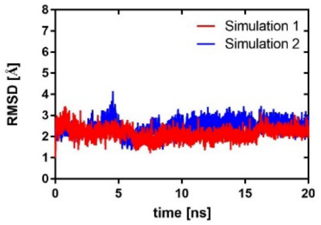
**Hit 922**	0.691 ± 0.051	0.837 ± 0.080	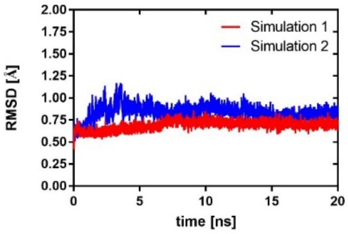	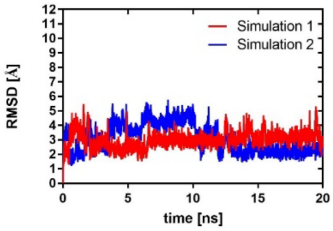
**GRL 0617**	2.166 ± 0.399	2.478 ± 0.165	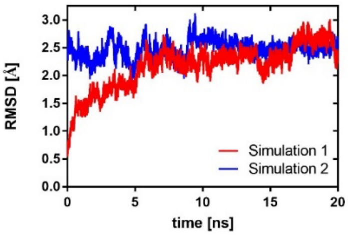	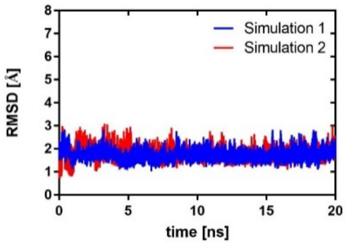
**Hit 826**	1.302 ± 0.131	1.280 ± 0.110	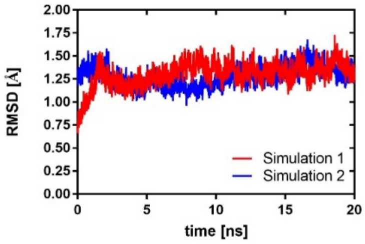	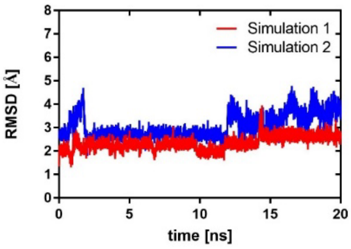
**Hit 372**	1.276 ± 0.144	1.369 ± 0.099	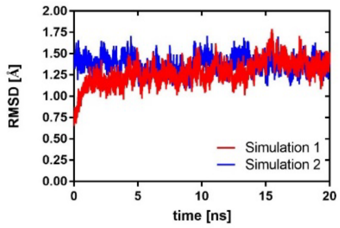	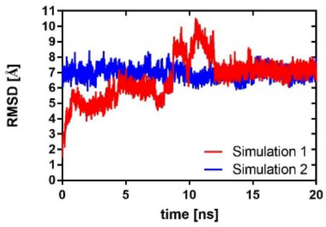

Table S5: Oligonucleotide sequences used in this study.

DNA primer	Sequence (from 5′ to 3′)
P1	gtttaactttaagaaggagatatacatatggaagtgaggactattaaggtgtttacaacag
P2	ctcagtggtggtggtggtggtgctcgagttttatggttgttgtgtaactgttttctttgtagaaaac
P3	ctcgagcaccaccaccac
P4	cagttattacccattgaaaaaggaagagtctgcaggcatcgtggtgtcac
P5	caggaagattgtataagcaaatatttctgcagcaatggcaacaacgttgcgc
P6	catatgtatatctccttcttaaagttaaacaaaattatttctagaggg
P7	gacgagcgtgacaccacgatgcctgcagactcttccttttcaatgggtaataactg
P8	gttgttgccattgctgcagaaatatttgcttatacaatcttcctgtttttggg
P9	gtttaactttaagaaggagatatacatatggaagtgaggactattaaggtgtttacaacag
P10	aaaacatatgtatatctccttcttaaagttaaacaaaattatttctagaggg
